# Relapsing peritoneal dialysis‑associated peritonitis caused by *Pseudomonas oryzihabitans* with concurrent tunnel infection: a case report and literature review

**DOI:** 10.1007/s13730-025-01044-8

**Published:** 2026-01-06

**Authors:** Maria Yoshida, Yujiro Maeoka, Minako Yoshida, Naoki Ishiuchi, Shunji Suemaru, Hiroshi Watanabe, Takao Masaki

**Affiliations:** 1 Department of Nephrology, Hiroshima University Hospital, 1-2-3 Kasumi, Minami-ku, Hiroshima, 734‑8551 Japan; 2 Department of Internal Medicine, Sanyo Hospital, 2-8-2, Nogami-cho, Fukuyama, 720-0815 Japan; 3 Department of General Surgery, Sanyo Hospital, 2-8-2, Nogami-cho, Fukuyama, 720-0815 Japan

**Keywords:** PD-associated peritonitis, *Pseudomonas oryzihabitans*, Opportunistic pathogen

## Abstract

Peritoneal dialysis (PD)-associated peritonitis is linked to an increased risk of catheter removal and PD discontinuation, with these risks occurring more frequently in relapsing peritonitis than in primary peritonitis. Relapses are often attributed to persistent infection originating from the PD catheter, including tunnel infection (TI). Although *Pseudomonas oryzihabitans* is a commensal bacterium and recognized opportunistic pathogen, only a limited number of cases of *P. oryzihabitans* peritonitis—and none involving relapsing peritonitis with concurrent TI—have been reported. A 73-year-old Japanese man undergoing PD presented with abdominal pain, fatigue, fever, and decreased PD fluid drainage. He was diagnosed with PD-associated peritonitis caused by *P. oryzihabitans*, which was preceded by a persistent exit-site infection due to different organisms. A 21-day course of antimicrobial therapy combined with peritoneal lavage led to clinical improvement. However, 27 days after completing treatment, he developed relapsing peritonitis due to *P. oryzihabitans*, complicated by TI suspected on computed tomography. However, the patient declined catheter removal and chose outpatient intraperitoneal antibiotics. The relapse resolved, but persistent yellow drainage prompted cuff-shaving surgery. We present a case of peritonitis caused by *P. oryzihabitans*, initially presenting with exit site infection and later complicated by TI at recurrence, that was successfully treated without PD catheter removal. This case suggests that cuff-shaving combined with appropriate antimicrobial therapy may be an effective treatment option for *P. oryzihabitans* peritonitis when the peritonitis and catheter infection are not caused by the same organism.

## Introduction

Peritoneal dialysis (PD)-associated peritonitis is a frequent complication of PD, contributing significantly to increased healthcare costs and mortality rates [1–3]. It often causes reduced peritoneal ultrafiltration capacity and is among the leading causes of permanent transfer to hemodialysis [4]. Relapsing peritonitis—defined by the International Society for Peritoneal Dialysis (ISPD) as an episode occurring within 4 weeks of completing therapy for a prior episode caused by the same organism—occurs in 5% to 20% of cases across various adult and pediatric series [5, 6]. In relapsing peritonitis, the risks of catheter removal and permanent transfer to hemodialysis are higher than those in uncomplicated or primary peritonitis [7]. These relapsing episodes may result not only from antimicrobial resistance but from persistent infection originating from the PD catheter, such as biofilm formation or tunnel infection (TI) [8].

Effective management of PD-associated peritonitis depends on accurate identification of causative organisms in the PD effluent [9]. Gram-positive cocci are the most frequently identified pathogens responsible for PD-associated peritonitis [10, 11]. These microorganisms typically originate from exogenous sources such as touch contamination, exit site infection (ESI) or TI, and catheter insertion procedures [12]. *Pseudomonas oryzihabitans* is a glucose non-fermenting gram-negative rod and a commensal bacterium found in moist environments such as soil [13–15]. Although rarely isolated in peritonitis, this pathogen is known to cause opportunistic infections including bacteremia, peritonitis, endophthalmitis, and cellulitis. These infections occur predominantly in patients receiving immunosuppressants, those with hematologic malignancies [14, 16, 17], and those undergoing catheterization [18–20]. To date, eight cases of PD-associated peritonitis caused by *P. oryzihabitans* have been reported, including one case of relapsing peritonitis. However, there have been no reported cases involving concurrent ESI or TI at the time of peritonitis diagnosis.

We herein report a rare case of peritonitis caused by *P. oryzihabitans*, presenting concurrently with ESI during the initial episode and TI during relapse, both due to other pathogens. At the time of relapse, the patient underwent appropriate antimicrobial therapy and cuff-shaving surgery to remove viscous fluid surrounding the catheter, effectively treating both the peritonitis and TI without removing PD catheter.

## Case report

A 73-year-old Japanese man undergoing PD for 14 months with end-stage kidney disease (ESKD) due to chronic glomerulonephritis, was admitted to our hospital with abdominal pain, fatigue, fever, decreased PD fluid removal, and cloudy PD effluent for 3 days. His medical history included hypertension and dyslipidemia, but not diabetes mellitus. He regularly took olmesartan, amlodipine, alfacalcidol, rosuvastatin, and febuxostat. He had recurrent purulent drainage at the exit site treated with cephalexin 8, 3, and 2 months before presentation. One month prior to admission, similar purulent discharge was observed, and cultures revealed coagulase-negative staphylococci (CNS) and gram-positive rods (GPR). To treat the ESIs, oral amoxicillin–clavulanic acid and topical sodium fusidic acid were administered.

**Fig. 1:**
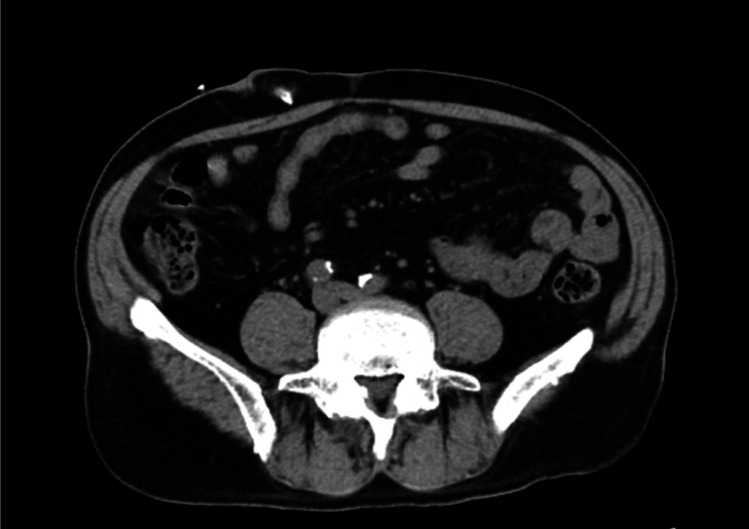
Plain computed tomography (CT) imaging of the first episode of peritonitis

**Fig. 2:**
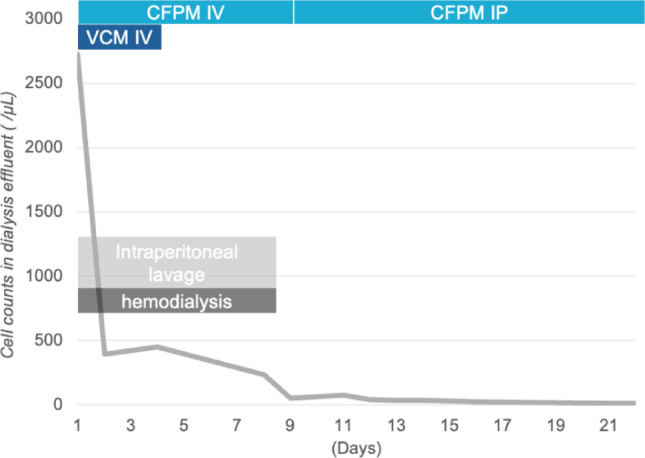
Clinical course during the first episode of peritonitis. Changes in the white cell count in the PD effluent over time are shown. CFPM, cefepime; VCM, vancomycin; IV, intravenous injection; IP, intraperitoneal injection

On admission, the patient was 162.8 cm tall and weighed 71.2 kg, with a 4-kg increase attributed to decreased PD fluid removal. His blood pressure was 158/86 mmHg, heart rate was 108 beats per minute, and body temperature was 37.2 °C. Physical examination revealed a flat and soft abdomen without signs of peritoneal irritation. Although there were no signs suggestive of TI, purulent yellow drainage was observed at the exit site. His white blood cell (WBC) count was 8,170/μL, and his serum C-reactive protein was mildly elevated at 2.27 mg/dL (Table 1). The PD effluent appeared cloudy and markedly yellow, with an elevated white cell count of 2725/μL and a predominance of neutrophils (88.0%). Plain computed tomography (CT) imaging showed increased density of intra-abdominal adipose tissue—a typical finding of PD-associated peritonitis—without signs of enteral infection (Fig. 1). Based on these findings, he was diagnosed with PD-related peritonitis. Empirical intravenous antimicrobial therapy was initiated on the day of presentation with cefepime (CFPM) at 0.5 g/day and vancomycin (VCM) at 1.0 g/day (Fig. 2). Hemodialysis was performed via an intravenous catheter along with intraperitoneal lavage due to both an increased cardiothoracic ratio and weight gain. The white cell count in the PD effluent gradually, decreased to 450/μL by day 4 (Fig. 2). Although blood cultures were negative, *P. oryzihabitans* was identified in the dialysis effluent using a mass spectrometer system (MALDI Biotyper system; Bruker Daltonics GmbH & Co. KG, Bremen, Germany) (Table 1). VCM was discontinued according to the results of the antibiotic susceptibility test (Table 2). On day 7, PD was resumed, following the white cell count dropped below 100/µL on day 9 and the patient was transitioned to outpatient treatment with intraperitoneal CFPM. A 21-days of antibiotic therapy resulted in a favorable clinical course and normalization of the exit site. The white cell count normalized by day 23, and antibiotic therapy was completed.

**Table 1:** Laboratory results on first admission

Parameter	Value	(Normal range)
*Blood*		
White cell count (/μL)	8170	(3300–8600)
Neutrophil (%)	77	(41.2–74.7)
Lymphocyte (%)	14.4	(21.3–50.3)
Monocyte (%)	6.5	(3.1–8.0)
Eosinophil (%)	2.1	(0.2–8.4)
Basophil (%)	2.1	(0.2–1.8)
Red blood cell (10^4^/μL)	405	(378–499)
Hemoglobin (g/dL)	12.5	(10.8–14.9)
Hematocrit (%)	38.1	(35.6–45.4)
Platelet (10^4^/μL)	22.4	(15.0–36.0)
Aspartate transaminase (U/L)	18	(13–33)
Alanine transaminase (U/L)	19	(8–42)
Lactate dehydrogenase (U/L)	285	(124–222)
γ-Glutamyltransferase (U/L)	39	(13–64)
Total bilirubin (mg/dL)	0.7	(0.4–1.5)
Serum albumin (g/dL)	4.5	(4.0–5.0)
Blood urea nitrogen (mg/dL)	44.3	(8–20)
Creatinine (mg/dL)	8.91	(040–0.70)
Na (mmol/L)	140	(138–146)
K (mmol/L)	4.4	(3.6–4.9)
Cl (mmol/L)	100	(99–109)
Calcium (mg/dL)	9.4	(8.6–10.4)
Phosphate (mg/dL)	3.8	(2.5–4.7)
Uric acid (mg/dL)	3.5	(2.3–7.0)
C-reactive protein (mg/dL)	2.27	(< 0.20)
*Peritoneal dialysis effluent*		
White cell count (/μL)	2725	Negative
Neutrophil (%)	88	Negative
Lymphocyte (%)	3	Negative
Lactate dehydrogenase (U/L)	46	
Serum albumin (g/dL)	0.3	
Amylase (IU/L)	16	
*Culture*		
Bacterial culture (PD effluent)	*Pseudomonas oryzihabitans*	Negative
Mycobacterium culture (PD effluent)	No data	Negative
Blood	Negative	Negative

**Table 2:** Antibiotic susceptibility test results of *Pseudomonas oryzihabitans* on first and second admissions

Antibiotic	The first admission		The second admission	
	MIC (µg/mL)	Susceptibility	MIC (µg/mL)	Susceptibility
Piperacillin	≦ 8	S	≦ 8	S
Ceftazidime	≦ 2	S	≦ 2	S
Ceftriaxone	4	S	≦ 2	S
Cefepime	≦ 2	S	≦ 2	S
Cefozopran	≦ 2	S	≦ 2	S
Imipenem	≦ 1	S	≦ 1	S
Meropenem	≦ 1	S	≦ 1	S
Doripenem	≦ 0.5	S	≦ 0.5	S
Biapenem	≦ 1	S	≦ 1	S
Aztreonam	> 16	R	16	I
Ampicillin/Sulbactam	16	I	16	I
Cefoperazone/Sulbactam	≦ 16	S	≦ 16	S
Tazobactam/Piperacillin	≦ 8	S	≦ 8	S
Gentamicin	≦ 2	S	≦ 2	S
Tobramycin	≦ 2	S	≦ 2	S
Amikacin	≦ 4	S	≦ 4	S
Isepamicin	≦ 4	S	≦ 4	S
Minocycline	≦ 4	S	≦ 4	S
Fosfomycin	≦ 64	S	≦ 64	S
Ciprofloxacin	≦ 0.25	S	≦ 0.25	S
Tosufloxacin	≦ 0.5	S	≦ 0.5	S
Levofloxacin	≦ 2	S	≦ 2	S
Pazufloxacin	≦ 2	S	≦ 2	S
Garenoxacin	≦ 4	S	≦ 4	S
Sitafloxacin	≦ 2	S	≦ 2	S
Sulfamethoxazole /Trimethoprim	> 40	R	≦ 40	S

MIC, minimal inhibitory concentration; S, susceptible; I, intermediate

**Fig. 3:**
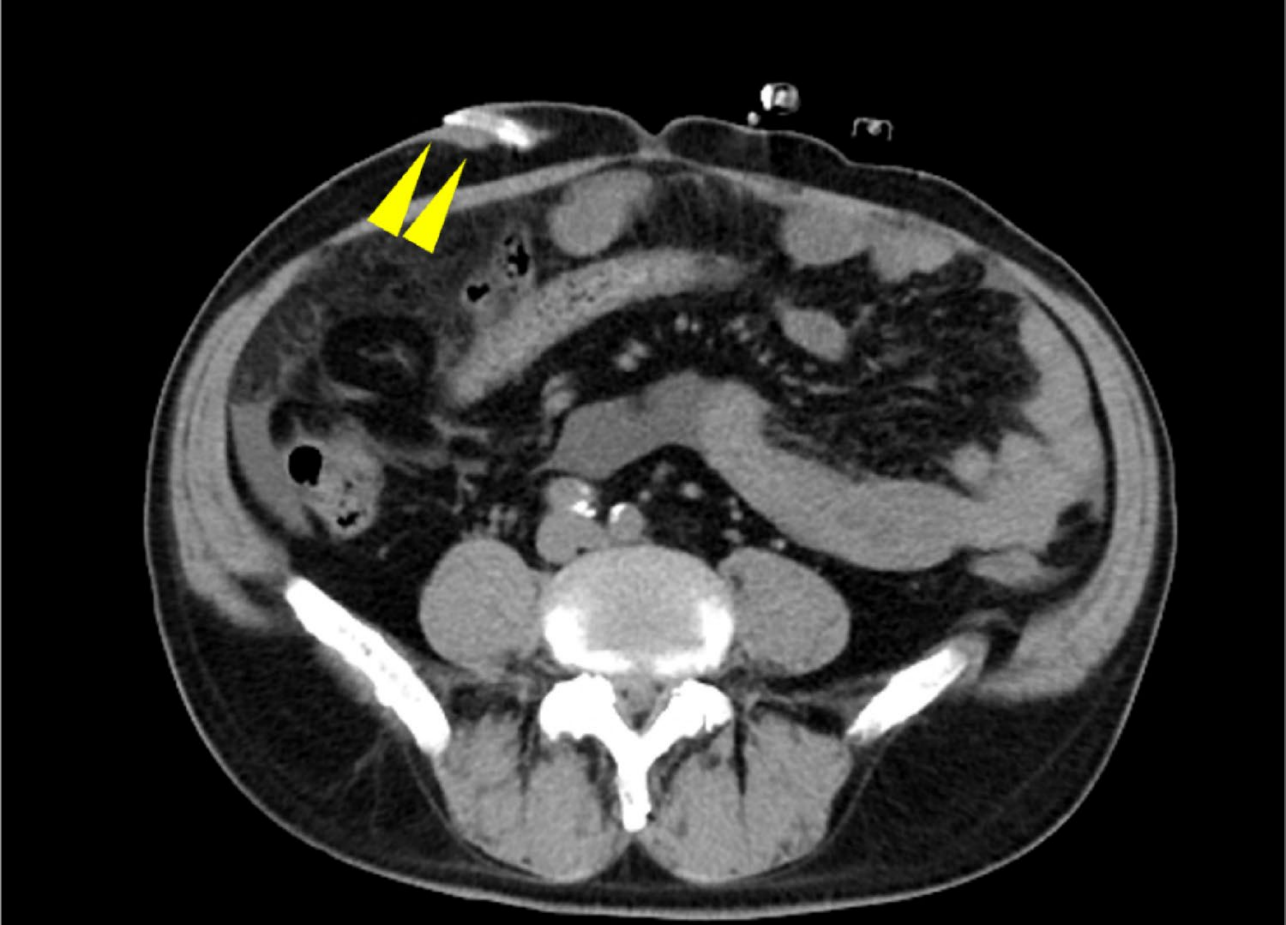
Plain computed tomography (CT) imaging of the second episode of peritonitis. The yellow arrows show chronic inflammation around the catheter near the external cuff

**Fig. 4:**
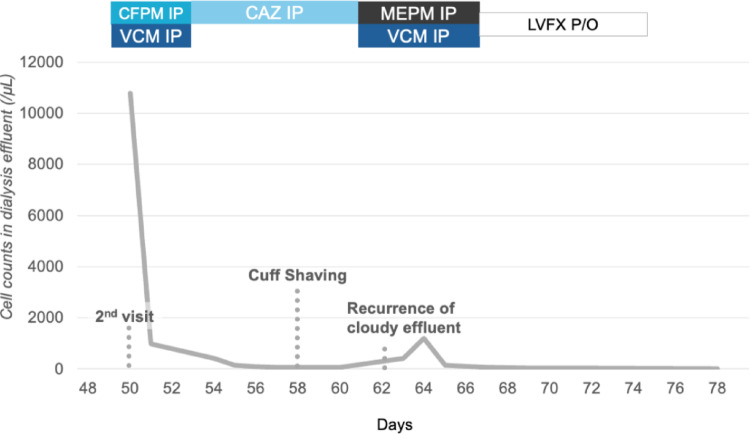
Clinical course during the second episode of peritonitis. Changes in the white cell count in the PD effluent (gray line) over time are shown. CFPM, cefepime; CAZ, ceftazidime; MEPM, meropenem; VCM, vancomycin; IP, intraperitoneal injection; LVFX, levofloxacin; P/O, per os (oral administration)

**Fig. 5:**
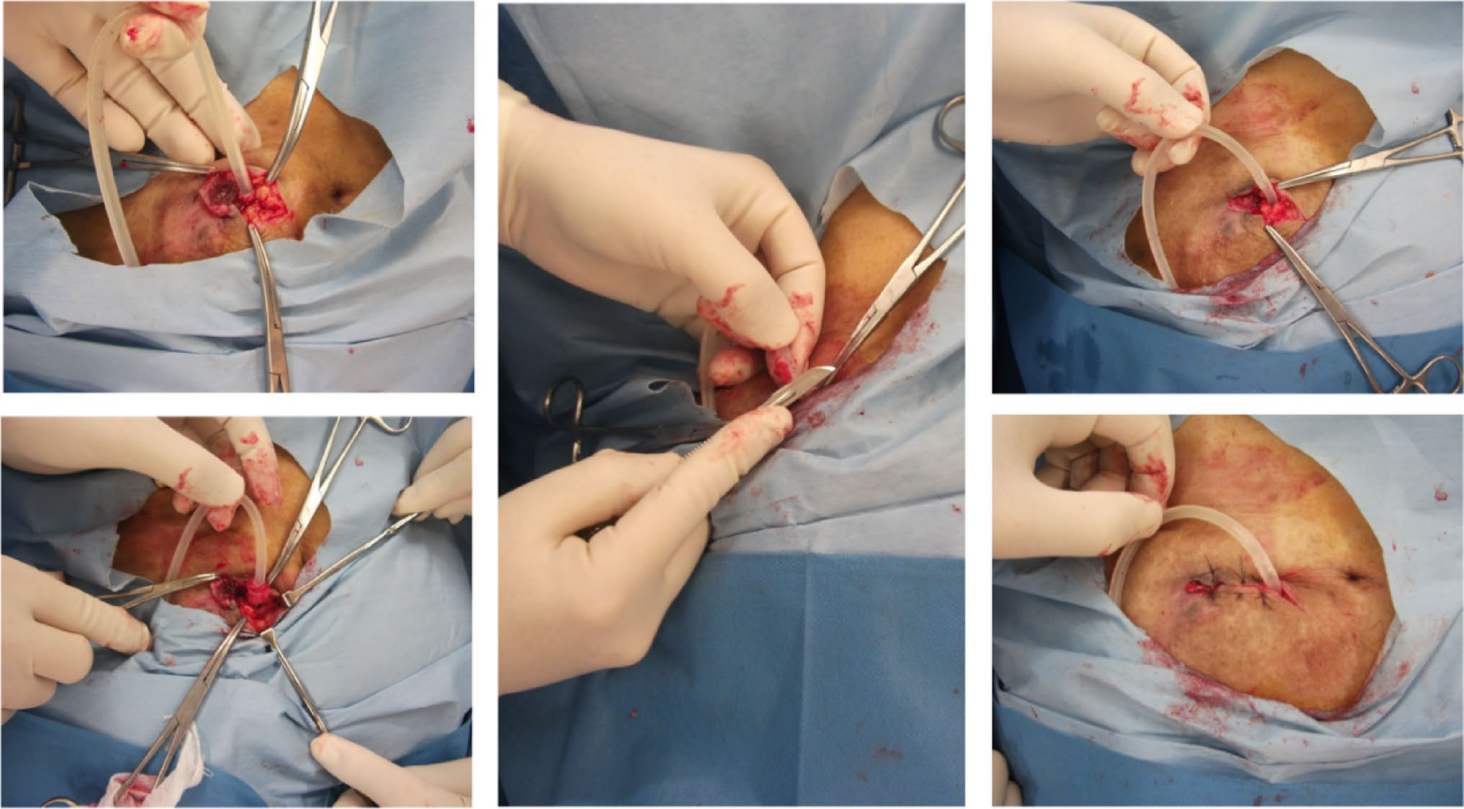
Intraoperative photographs of cuff-shaving surgery. Intraoperative records: Mild granulation tissue was observed at the exit site. A 5.0 cm skin incision was made toward the medial side, reaching the outer cuff located 3 cm from the exit site. Within a 2 cm range from the exit site, a viscous sheath was present around the catheter, and a bacterial culture was obtained. The outer cuff was adherent to the surrounding tissue, with no pus present. The external cuff was detached from the surrounding tissue, and the frayed edges of the cuff were trimmed with a No. 10 scalpel blade. The skin at the old exit site was trimmed, and a tunnel was created extending 1 cm inward from the external cuff. The area near the exit site was scraped with a sharp spoon, rinsed with saline, and the skin behind the catheter was partially closed with 3–0 nylon sutures using the vertical mattress technique. A straight catheter (J1; Hayashidera Medinol, Japan) was used. Shaving and unroofing were performed by the surgeon (H.Y) in the operating room, and there were no technical difficulties or delays

On day 49, the patient was readmitted to our hospital with decreased PD fluid removal and cloudy PD effluent. Although there was no purulent drainage or exudate at the exit site, yellow crusting was noted. No significant physical findings indicative of TI were observed, but CT imaging revealed chronic inflammation around the catheter near the external cuff (Fig. 3). Peritoneal fluid analysis showed an elevated white cell count with neutrophil predominance, and *P. oryzihabitans* was identified in the dialysate culture, confirming relapsing peritonitis. TI was clinically suspected; however, the patient declined both catheter removal and hemodialysis, opting instead for outpatient treatment with intraperitoneal antibiotics. The dialysate culture revealed an antimicrobial resistance pattern similar to that of the initial peritonitis (Table 2). Treatment began with intraperitoneal CFPM and VCM for the convenience of patients, later switched to ceftazidime (Fig. 4), which has a similar minimal inhibitory concentration to CFPM but a narrower antimicrobial spectrum. He was able to visit the hospital almost daily, where peritoneal dialysis fluid mixed with antibiotics was prepared and administered after confirming drainage clarity. Although white cell count in the PD effluent fell below 100/µL on day 57, yellow drainage at the exit site persisted, prompting cuff-shaving surgery. During the procedure, viscous fluid was found around the catheter within 2 cm of the exit site corresponding to the area seen on CT, although swab cultures of the fluid were negative. No pus was observed on the external cuff, and cuff-shaving was performed as planned (Fig. 5). Even after surgery, recurrence of cloudy effluent required continued antibiotics. Intraperitoneal administration of meropenem and VCM was initiated empirically to broaden antimicrobial spectrum and address the possibility of peritonitis caused by different organisms, followed by a switch to oral levofloxacin, led to resolution of cloudy effluent and restored fluid removal. The white cell count in the PD effluent dropped below 100/µL on day 67 and normalized by day 79. Postoperatively, the exit site remained healthy without purulent drainage.

## Discussion

*Pseudomonas oryzihabitans* is a glucose non-fermentative, gram-negative, rod-shaped aerobic bacillus and an environmental opportunistic pathogen commonly found in natural soil, water, and moist indoor environments [13–15]. This bacillus is relatively rarely isolated. Infections caused by this pathogen typically occur in immunocompromised patients, including those undergoing steroid therapy and those with hematologic malignancies [14, 16, 17]. Indwelling catheters are a major predisposing factor for *P. oryzihabitans* infections [18–20]. To date, PD-associated peritonitis caused by *P. oryzihabitans* has been rarely reported, and only in Europe and the United States [21–27] (Table 3). Therefore, to our knowledge, this is the first English-language case report from Asia.

**Table 3:** Clinical outcomes of previous cases of peritonitis caused by *Pseudomonas oryzihabitans*

Author	Year	Age	Sex	ESKD cause	Time on PD (mo)	Pervious episodes of PD-related infection	Species	Therapy	Outcome
Silver [21]	1985	54	M	CGN, Diabetes	20	+ (*A. calcoaceticus var. anitratus*, *E. coli*)	*P. oryzihabitans*	CEZ + TOB, TOB	Cure
Amber [22]	1987	47	M	Goodpasture’s syndrome	54	+ (CNS)	*P. oryzihabitans*	Cephapirin + GM, ABPC	Cure
Bendig [23]	1989	62	M	Obstructive uropathy	19	+ (CNS)	*P. oryzihabitans*	CXM + GM, GM	Cure
Esteban [24]	1993	63	M	Diabetes	14	+ (*S. aureus*)	*P. oryzihabitans*	VCM + TOB, TOB	Cure
Cusimano [25]	1997	44	F	N/A	N/A	+ (CNS, *S. aureus*)	*P. oryzihabitans*	VCM + TOB, TOB	Cure
Esteban [24]	2002	50	F	IgA nephropathy	38	+ (*E. coli*, *P. aeruginosa*)	*P. oryzihabitans*	VCM + GM, CXM + GM	Cure
Levitski-Heikkila [26]	2005	41	M	Hypertension	11	–	*A. radiobacter*,* P. oryzihabitans*	CEZ + GM	**Reccurent** *(C. aquaticum)*
Papakonstantinou [27]	2005	22	M	N/A	7	+ (*micrococcus*)	*P. oryzihabitans*	VCM + AMK, CFTX + AMK	**Relapsing**
Present case	2025	73	M	CGN	14	+ (CNS, gram-positive rods)	*P. oryzihabitans*	VCM + CFPM, CFPM	**Relapsing**

ESKD, end-stage kidney disease; CGN, chronic glomerulonephritis; CEZ, cefazolin; TOB, tobramycin; GM, gentamicin; CNS, coagulase-negative staphylococci; ABPC, ampicillin; CXM, cefuroxime; AMK, amikacin; CFTX, ceftriaxone; CFPM, cefepime. Recurrence or relapse cases are highlighted in bold.

Several cases of PD-associated peritonitis caused by *P. oryzihabitans* have been reported over different decades, with three cases in the 1980s–1990s [21–23], two in the 1990s [24, 25], and three in the 2000s [24, 26, 27] (Table 3). Previous studies have noted challenges in identifying *P. oryzihabitans* [15]. In contrast to these earlier cases, *P. oryzihabitans* in the present case was identified with high confidence at the species-level identification using the MALDI Biotyper system, which employs matrix-assisted laser desorption/ionization time-of-flight mass spectrometry (MALDI-TOF MS). This method may play an important role in achieving an accurate and reliable identification of opportunistic pathogens that were previously difficult to detect, such as *P. oryzihabitans*, in cases of peritonitis.

In line with previous findings, *P. oryzihabitans* infections primarily occur in patients with indwelling catheters as in this case and in immunocompromised individuals. Given this background, when this bacterium is isolated from clinical specimens, evaluation of the host’s condition is warranted [15]. A closer examination of prior studies on PD infections revealed no significant bias in the primary diseases leading to ESKD, age or sex. The onset of infection after initiating PD ranged from 7 to 54 months. Notably, PD-related infections caused by other pathogens and their treatments were reported in eight of nine cases prior to infection with *P. oryzihabitans*. Importantly, all episodes of peritonitis, including relapses, were successfully treated with appropriate antimicrobial therapy. This contrasts with non–PD-associated infections, which often require removal of foreign device [14]. These findings suggest that while *P. oryzihabitans* peritonitis is rare and frequently preceded by other PD-related infections, it can be effectively managed with timely and adequate antimicrobial treatment.

The 2012 ISPD guidelines allowed oral, intraperitoneal, or intravenous antibiotics for catheter tunnel infections, whereas the 2022 ISPD guidelines recommend intraperitoneal antibiotics as the preferred route for peritonitis, provided their compatibility and stability are ensured, unless the patient presents with systemic sepsis [2]. In this case, intravenous antibiotic therapy was initially selected due to insurance coverage limitations and limited institutional experience with intraperitoneal administration in the absence of a full-time nephrologist. At recurrence, the patient refused hospitalization; therefore, in collaboration with nephrologists, outpatient care with intraperitoneal administration was provided to achieve higher antibiotic concentration in the peritoneal cavity. According to the JSDT Renal Data Registry, intravenous administration alone is the most common route in Japan (29.5%), followed by combined intravenous and transperitoneal administration (26.4%), and transperitoneal administration alone (17.0%) [28]. In particularly facility with smaller PD populations, intravenous therapy remains practiced in a substantial proportion of PD-associated peritonitis cases.

In previous cases without ESI or TI, infection was thought to occur via an exogenous-transluminal route [26, 27] because *P. oryzihabitans* has been detected in contaminated household environments such as synthetic bath sponges [29]. By contrast, our patient experienced persistent ESI over several months during the first episode of PD-associated peritonitis caused by *P. oryzihabitans*. Additionally, at the onset of relapsing peritonitis, CT imaging revealed TI despite an intact exit site. Although *P. oryzihabitans* was not isolated from the cultures, the persistently moist local environment may have contributed to the development of peritonitis [22, 24]. While ESI is a known risk factor for PD-associated peritonitis, the causative organisms often differ from those isolated from preceding ESI [12]. In this case, the patient’s hand hygiene and disinfection practices and PD handling were appropriate. Furthermore, antimicrobial resistance was unlikely to be the cause of relapse because antibiotic susceptibility testing of *P. oryzihabitans* were similar in both episodes (Table 2). Therefore, we speculate that while the initial exit-site infection was due to CNS and GPR, a subsequent persistent local infiltrative environment may have contributed to both the onset and recurrence of infection with *P. oryzihabitans*. Nevertheless, the possibility of contamination via the exogenous-transluminal route cannot be entirely excluded.

Our patient had a recurrent catheter infection with TI, and catheter removal should have been considered as a treatment option, in accordance with the 2022 ISPD guidelines [2]. However, catheter removal was not possible because of patient’s refusal. As an alternative, cuff-shaving was performed as an outpatient procedure to treat chronic inflammation around the catheter, based on our speculation that the peritonitis and the catheter infection were not caused by the same organism. Subcutaneous pathway diversion was not performed simultaneously because of concern that persistent infection could spread to the internal cuff and lead to catheter-related infection. Persistent infection with this organism may relate to its biofilm-forming ability around the catheter [26]. Taken together, cuff-shaving combined with adequate antimicrobial therapy may have contributed to biofilm disruption and the resolution of peritonitis. Patients should be thoroughly informed at the initiation of PD that temporary catheter removal or PD discontinuation may become necessary in the event of infection. If catheter removal is not feasible because of lack of patient consent, local drainage and appropriate antimicrobial therapy may serve as an alternative treatment option.

A limitation of this case is that certain diagnostic tests were not performed, which might have provided further confirmation of the diagnosis. First, culture tests of the exit site were lacking during both the initial and the second peritonitis episodes. Second, ultrasound imaging of the suspected tunnel infection site was not available. Third, the postoperative recurrence of cloudy dialysate may have been due either to residual infection around the cuff that was not completely eradicated during surgery or to new touch contamination, although culture testing was not performed and the precise mechanism could not be determined.

In conclusion, we have reported a rare case of peritonitis caused by *P. oryzihabitans* in a patient with no apparent water exposure. While peritonitis caused by this bacillus may be effectively managed with timely and appropriate antibiotic therapy, concurrent TI may necessitate cuff-shaving or catheter removal.

## References

[1] Brown MC, Simpson K, Kerssens JJ, Mactier RA. Scottish renal registry. Peritoneal dialysis-associated peritonitis rates and outcomes in a national cohort are not improving in the post-millennium (2000-2007). Perit Dial Int. 2011;31:639–50. 21804138 10.3747/pdi.2010.00185

[2] Li PK, Chow KM, Cho Y, Fan S, Figueiredo AE, Harris T, et al. ISPD peritonitis guideline recommendations: 2022 update on prevention and treatment. Perit Dial Int. 2022;42:110–53. 35264029 10.1177/08968608221080586

[3] Cho Y, Johnson DW. Peritoneal dialysis-related peritonitis: towards improving evidence, practices, and outcomes. Am J Kidney Dis. 2014;64:278–89. 24751170 10.1053/j.ajkd.2014.02.025

[4] Htay H, Cho Y, Pascoe EM, Darssan D, Nadeau-Fredette AC, Hawley C, et al. Multicenter registry analysis of center characteristics associated with technique failure in patients on incident peritoneal dialysis. Clin J Am Soc Nephrol. 2017;12:1090–9. 28637862 10.2215/CJN.12321216 PMC5498362

[5] Kavanagh D, Prescott GJ, Mactier RA. Peritoneal dialysis-associated peritonitis in Scotland (1999-2002). Nephrol Dial Transplant. 2004;19:2584–91. 15304559 10.1093/ndt/gfh386

[6] Schaefer F, Feneberg R, Aksu N, Donmez O, Sadikoglu B, Alexander SR, et al. Worldwide variation of dialysis-associated peritonitis in children. Kidney Int. 2007;72:1374–9. 17882152 10.1038/sj.ki.5002523

[7] Burke M, Hawley CM, Badve SV, McDonald SP, Brown FG, Boudville N, et al. Relapsing and recurrent peritoneal dialysis-associated peritonitis: a multicenter registry study. Am J Kidney Dis. 2011;58:429–36. 21601333 10.1053/j.ajkd.2011.03.022

[8] Szeto CC, Kwan BC, Chow KM, Law MC, Pang WF, Chung KY, et al. Recurrent and relapsing peritonitis: causative organisms and response to treatment. Am J Kidney Dis. 2009;54:702–10. 19577352 10.1053/j.ajkd.2009.04.032

[9] Kanjanabuch T, Puapatanakul P, Saejew T, Pavatung P, Manuprasert W, Leelahavanichkul A, et al. The culture from peritoneal dialysis catheter enhances yield of microorganism identification in peritoneal dialysis-related peritonitis. Perit Dial Int. 2020;40:93–5. 32063145 10.1177/0896860819878387

[10] Mizuno M, Ito Y, Tanaka A, Suzuki Y, Hiramatsu H, Watanabe M, et al. Matsuo S peritonitis is still an important factor for withdrawal from peritoneal dialysis therapy in the Tokai area of Japan. Clin Exp Nephrol. 2011;15:727–37. 21691738 10.1007/s10157-011-0471-8

[11] Prasad KN, Singh K, Rizwan A, Mishra P, Tiwari D, Prasad N, et al. Microbiology and outcomes of peritonitis in northern India. Perit Dial Int. 2014;34:188–94. 24584592 10.3747/pdi.2012.00233 PMC3968104

[12] van Diepen AT, Tomlinson GA, Jassal SV. The association between exit site infection and subsequent peritonitis among peritoneal dialysis patients. Clin J Am Soc Nephrol. 2012;7:1266–71. 22745277 10.2215/CJN.00980112 PMC3408122

[13] Rahav G, Simhon A, Mattan Y, Moses AE, Sacks T. Infections with *Chryseomonas luteola* (CDC group Ve-1) and *flavimonas oryzihabitans* (CDC group Ve-2). Medicine (Baltimore). 1995;74:83–8. 7891546 10.1097/00005792-199503000-00003

[14] Lin RD, Hsueh PR, Chang JC, Teng LJ, Chang SC, Ho SW, et al. *Flavimonas oryzihabitans* bacteremia: clinical features and microbiological characteristics of isolates. Clin Infect Dis. 1997;24:867–73. 9142784 10.1093/clinids/24.5.867

[15] Nei T, Sonobe K, Onodera A, Itabashi T, Yamaguchi H, Maeda M, et al. Two cases with bacteremia suspected to be due to relatively rare *Pseudomonas* (*Flavimonas*) *oryzihabitans*. J Infect Chemother. 2015;21:751–5. 26184853 10.1016/j.jiac.2015.06.005

[16] Boeckh M, Boivin G. Quantitation of cytomegalovirus: methodologic aspects and clinical applications. Clin Microbiol Rev. 1998;11:533–54. 9665982 10.1128/cmr.11.3.533 PMC88895

[17] Giacometti A, Cirioni O, Quarta M, Schimizzi AM, Del Prete MS, Scalise G. Unusual clinical presentation of infection due to *Flavimonas oryzihabitans*. Eur J Clin Microbiol Infect Dis. 1998;17:645–8. 9832267 10.1007/BF01708348

[18] Romanyk J, Gonzalez-Palacios R, Nieto A. A new case of bacteraemia due to *Flavimonas oryzihabitans*. J Hosp Infect. 1995;29:236–7. 7615943 10.1016/0195-6701(95)90336-4

[19] Reed RP. *Flavimonas oryzihabitans* sepsis in children. Clin Infect Dis. 1996;22:733–4. 8729230 10.1093/clinids/22.4.733

[20] Qian K, Wang S. Infections caused by *Flavimonas oryzihabitans*. Chin Med J (Engl). 2001;114:394–8. 11780462

[21] Silver MR, Felegie TP, Sorkin MI. Unusual bacterium, group Ve-2, causing peritonitis in a patient on continuous ambulatory peritoneal dialysis. J Clin Microbiol. 1985;21:838–9. 3998117 10.1128/jcm.21.5.838-839.1985 PMC271793

[22] Amber IJ, Reimer LG. *Pseudomonas* sp. group Ve-2 bacterial peritonitis in a patient on continuous ambulatory peritoneal dialysis. J Clin Microbiol. 1987;25:744–5. 3571484 10.1128/jcm.25.4.744-745.1987 PMC266075

[23] Bendig JW, Mayes PJ, Eyers DE, Holmes B, Chin TT. *Flavimonas oryzihabitans* (*Pseudomonas oryzihabitans*; CDC group Ve-2): an emerging pathogen in peritonitis related to continuous ambulatory peritoneal dialysis? J Clin Microbiol. 1989;27:217–8. 2913032 10.1128/jcm.27.1.217-218.1989 PMC267271

[24] Esteban J, Martin J, Ortiz A, Santos-O’Connor F, Cabria F, Reyero A. *Pseudomonas oryzihabitans* peritonitis in a patient on continuous ambulatory peritoneal dialysis. Clin Microbiol Infect. 2002;8:607–8. 12427223 10.1046/j.1469-0691.2002.00447.x

[25] Cusimano A, Husserl FE. *Flavimonas oryzihabitans* peritonitis in CAPD. Perit Dial Int. 1997;17:406–7. 9284477

[26] Levitski-Heikkila TV, Ullian ME. Peritonitis with multiple rare environmental bacteria in a patient receiving long-term peritoneal dialysis. Am J Kidney Dis. 2005;46:e119–24. 16310563 10.1053/j.ajkd.2005.08.021

[27] Papakonstantinou S, Dounousi E, Ioannou K, Tsouchnikas I, Kelesidis A, Kotzadamis N, et al. A rare cause of peritonitis caused by *Flavimonas oryzihabitans* in continuous ambulatory peritoneal dialysis (CAPD). Int Urol Nephrol. 2005;37:433–6. 16142579 10.1007/s11255-004-4650-3

[28] Hanafusa N, Abe M, Joki N, Ogawa T, Kanda E, Kikuchi K, et al. Annual dialysis data report 2019, JSDT Renal Data Registry. Ren Replace Ther. 2023;9:47.

[29] Marín M, de García Viedma D, Martín-Rabadán P, Rodríguez-Créixems M, Bouza E. Infection of hickman catheter by *Pseudomonas* (formerly flavimonas) oryzihabitans traced to a synthetic bath sponge. J Clin Microbiol. 2000;38:4577–9. 11101598 10.1128/jcm.38.12.4577-4579.2000 PMC87639

